# Sanctioning of Illegal and Dangerous Ruck Cleanouts During the 2018 Super Rugby Competition

**DOI:** 10.3389/fpsyg.2019.00803

**Published:** 2019-04-05

**Authors:** Wilbur Kraak, Jenna Bam, Stephanie Kruger, Stephanie Henderson, Ugan Josias, Keith Stokes

**Affiliations:** ^1^Department of Sports Science, Faculty of Medicine and Health Sciences, Stellenbosch University, Stellenbosch, South Africa; ^2^Department for Health, University of Bath, Bath, United Kingdom

**Keywords:** injury prevention, ruck cleanouts, sanctioning, dangerous play, referees, attack, defense

## Abstract

Rugby is a high contact sport that results in many injuries. The majority of injuries at senior elite levels result from contact phases during match-play. It is estimated that 10% of all match injuries are associated with the ruck in professional and community rugby. Surveillance of legal and illegal ruck cleanouts and the sanctions imposed by the on-field referees will help identify whether referees are actually enforcing the law according to the laws of the game, which will consequently contribute to the creation and implementation of further injury prevention strategies. Players should play the game in accordance with the laws of the game and be mindful of their own safety and that of others. Coaches and trainers of the game have the responsibility to ensure that players are prepared in a manner that comply with the World Rugby (WR) laws of the game and safe practices. Laws and law amendments are fundamental to the development of sport and introduced for a variety of reasons. The aim of this study was to investigate the rate of sanctioning of illegal and dangerous ruck cleanouts during the 2018 Super Rugby competition by using Nacsport Basic+ video software; 120 round robin matches from the 2018 Super Rugby competition were coded and analyzed. The analysis of the intra reliability showed an almost perfect (>0.95) agreement between all the performance indicators. In total, 22,281 ruck cleanouts were coded of which 9% (*n* = 2111) were illegal ruck cleanouts and 93% were not sanctioned by the referees; 57% (1087 out of 1953) of the illegal ruck cleanouts not sanctioned by the referees were deemed dangerous. The majority of dangerous illegal ruck cleanouts not sanctioned by the referees were “*shoulder charge*” (88%, *n* = 280), “*neck roll*” (86%, *n* = 100), and “contact above the shoulder” (81%, *n* = 201). To aid injury prevention efforts in rugby, future research studies should investigate why on-field referees are not sanctioning all illegal and dangerous ruck cleanouts according to WR *Laws of the Game*.

## Introduction

Rugby union (rugby) is an invasion field-based team sport. At senior level matches last 80 min, which are characterized by short intermittent bouts of high intensity activity and multiple high impact contact situations between 30 players (Fuller et al., 2007). Rugby also has one of the highest incidences of injury, irrespective of the injury definition used in the investigation (Fuller et al., 2010). The majority of injuries at senior and elite levels of rugby result from contact phases of play (Williams et al., 2013). More or less 10% of all match injuries are associated with the ruck in professional (Rugby Football Union, 2018) and community rugby (Roberts et al., 2015).

Any sport involving physical contact such as rugby has inherent risks toward its players. Players are required to play the game in accordance with the WR *Laws of the Game* and should be mindful of their own safety and that of their own and opposition players. It is the responsibility of the coaches and trainers to ensure that players are prepared in a manner that comply with the laws of the game and safe practices (Kraak et al., 2016, 2017). The on-field referees are responsible for interpreting and applying the laws of the game during the match, thereby protecting the players from potential injury risk. Apart from the maintenance of “fair play,” the decisions of referees can affect the outcome of a game significantly (Mascarenhas et al., 2005; Spitz et al., 2016). Laws and law amendments are fundamental to the development of a sport and introduced for a variety of reasons (Kraak and Welman, 2014). Some of the reasons why law changes and experimental law variations are implemented in rugby are in response to player performance, to ensure safety of all role-players involve, increase participation and enjoyment, promote continuity of rugby, technological advancement, and commercial pressures, as well as to retain game integrity and development (Eaves et al., 2008). World Rugby (WR) replacing the International Rugby Board (IRB) in 2014 is responsible for delivering safe, enjoyable, and entertaining rugby tournaments and events. Therefore, WR *Laws of the Game* provide a framework by which WR ensures these aspects (Murray et al., 2014) are achieved.

Injury prevention programs such as WR’s Rugby Ready, New Zealand’s RugbySmart, Australia’s Smart Rugby, and South Africa’s BokSmart direct their interventions and programs toward coach and trainer (will hopefully be transferred to the player) and referee education, because these role-players have a large influence on player behavior during training and match play. These injury prevention programs are put in place by various governing bodies to assess and educate coaches (players through the coaches) and referees on the prevalence of injury and how to overcome them, in order to make rugby as safe as possible for its players (Viljoen and Patricios, 2012; Roberts et al., 2015). Specifically, coaches can reduce injury risk through coaching better techniques during training and referees by applying the WR *Laws of the Game* and in the context of this study, penalizing illegal ruck cleanouts during matches. A study by Kraak et al. (2016) indicates that because of law changes the number of ball carriers increased from 184 to 219 in the Super Rugby tournament between 2008 and 2013. Hendricks et al. (2018) indicate that 65% of all collisions (ball carrier and defender engaged in a tackle), resulted in rucks in professional rugby. Rucks are one of the most frequently occurring contact events in rugby during match play. The ruck is a contest between the attacking and defending team and players involved in the ruck need to be on their feet. A cleanout is an action by the arriving players to either retain (attacking team) or regain (defending team) possession. The ability to repeatedly engage and win rucks has been associated with team success. Ortega et al. (2009) and Kraak and Welman (2014) state that winning teams regained ball possession at rucks more frequently than losing teams in the Six Nations competition. For the attacking team to retain possession and for the defending team to try to regain possession at the ruck, the teams will have to utilize specific clean out techniques. Ruck clean out techniques used by players during matches could often be deemed illegal according to the WR *Laws of the Game*, and could be considered dangerous in many cases. Illegal ruck clean out types according to the 2018 WR *Laws of the Game* (Law 9.20 and 15.5-9), include: (a) neck roll; (b) shoulder charge; (c) contact above the shoulder of an opposition player; (d) side entry; (e) not grasping onto team mate when cleaning; (f) not supporting own body weight; (g) clean out a player not involved in the ruck; and (h) joining the ruck, while in an offside position. The minimum sanction for all of these infringements is a penalty kick for the opposition. It should be noted that not all illegal ruck clean out techniques are deemed dangerous with a potential injury risk.

According to Van Mechelen and Hlobil (1992), the first step in injury prevention process is the surveillance step. A recent study by Brown et al. (2018) investigated the sanctioning of illegal tackling in the South African under 18 Craven Week rugby tournament and revealed that 59% of illegal tackles were not penalized appropriately by the referees. A study by Fuller et al. (2010) on professional English rugby found that only a minority of illegal/dangerous tackles were penalized correctly by the on-field referee according to the laws of the game; 6% (14 out of 238) of the high tackles (to the head/neck region of ball carriers) were penalized by the on-field referees in the study of Fuller et al. (2010). Surveillance of legal and illegal (dangerous and not-dangerous) ruck clean outs and the sanctions imposed by referees will help identify whether the referees are actually enforcing the laws according to the WR law book. The findings of the current study can lead to the development and implementation of further injury prevention strategies in order to make the game safer for all the role-players involved. Therefore, the primary aim of this study was to investigate the rate of sanctioning of illegal and dangerous ruck cleanouts during the 2018 Super Rugby competition.

## Materials and Methods

### Research Design

The study followed a descriptive and retrospective research design. Ethical approval (SU-HSD-001220) was obtained from the Research Ethics Committee: Human Research at Stellenbosch University.

### Sample

Televised video recordings of (*N* = 120) round robin matches from the 2018 Super Rugby competition were used for this study. The Super Rugby competition is a professional men’s rugby competition involving teams from Argentina, Australia, New Zealand, South Africa, and Japan. The Western Province Rugby Union (South Africa) video analysis department supplied the video recordings.

### Data Collection Procedure

#### Coding

Nacsport software (version: Basic+, Spain, 2008) was used to code all ruck clean outs of the 120 round robin matches. Prior to coding, a “*gold standard*” was set by an international referee, using the 2018 WR *Laws of the Game* definitions and analyzing a match in conjunction with the coder that consisted of 180 legal and illegal clean-outs. The performance indicators displayed in Table 1 were analyzed based on the aims of the study. The software allowed control over the speed at which each activity could be viewed, and the recording and saving of each coded event into a database thereafter, reflected as performance indicators.

**Table 1:** Performance indicators and operational definitions used in the study.

Performance indicators	Operational definition
Ruck World Rugby (2018)	The ruck is defined as a phase of play where one or more players from each team remain on their feet and are in physical contact close around the ball on the ground
Attacking team cleaning Hendricks et al. (2018)	Attackers are actively driving opponents off the ball in order to retain possession
Defending team cleaning Hendricks et al. (2018)	Defenders are actively attempting to regain possession
Match period Brown et al., 2018	Each match was divided into two halves of 40 min (first and second half) and four quarters of 20 min (first, second, third, and fourth quarter)
Arrival at the ruck: attacking and defending team Hendricks et al. (2018)	first, second, third, and fourth cleaner
Types of illegal ruck clean outs World Rugby (2018)	– *Neck roll*: A cleaner must not grasp an opposition player around the neck area to clean out – *Not supporting own body weight*: A player cleaning out a ruck must be on his feet – *Joining the ruck while in an offside position*: A player cleaning at the ruck may not do so while in an offside position. Non-participants at the breakdown must be behind the hindmost foot of the last player in their side of the ruck – *Shoulder charge*: A player must not charge into a ruck. Charging includes any contact made without use of the arms, or without grasping a player – *Side entry*: A cleaner must join alongside but not in front of the hindmost player – *Not grasping on teammate when cleaning*: A player joining a ruck must bind on a teammate or an opponent, using the whole arm. The bind must either precede, or be simultaneous with, contact with any other part of the body of the player joining the ruck – *Cleaning a player not involved in the ruck*: A cleaner must not take out opposition players who are not part of the ruck – *Contact above shoulder of opposition player*: A cleaner must not make contact with an opponent above the line of the shoulders
Sanctioning of illegal ruck clean outs World Rugby (2018)	Whether the illegal ruck cleanout was sanctioned (the first infringement) or not by the referee (s) according to the World Rugby *Laws of the Game*
Dangerous clean out	Dangerous clean out – action was deemed dangerous if the action of the player could lead to possible injury of (a) himself, (b) own players, and (c) opposition players


#### Reliability

The reliability of the coded ruck cleanouts was tested using inter-rater reliability. The test and retest reliability of coded performance indicators were assessed using methods described by O’Donoghue (2010). In brief, performance indicators and operational definitions were developed based on published peer-reviewed literature specifically pertaining to the ruck area and clean outs and the 2018 WR *Laws of the Game*. After all the matches were coded by the primary researcher, an international referee and rugby injury specialist re-coded 25% (*n* = 30) of the matches which were randomly selected by the statistician in order to test for the inter-rater reliability. The inter-rater reliability of the coding was determined by using the intraclass correlation coefficient (ICC) of the test and retest data (Gratton and Jones, 2004). The inter-rater agreement data was interpreted as follows: poor (<0.20), fair (0.30–0.40); moderate (0.50–0.60); strong (0.70–0.95); and almost perfect (>0.95). The test and retest data showed that the agreement between all the performance indicators was almost perfect (Table 2), and thus considered as very reliable and were included in the study.

**Table 2:** Inter-rater reliability of the coding test-retest using intraclass correlation coefficient (ICC).

Reliability	Ruck clean out	Illegal ruck clean out	Illegal ruck clean out sanctioned	Illegal ruck clean out not sanctioned	Dangerous illegal ruck clean out not sanctioned
Inter-reliability	1.00	0.98	1.00	0.97	0.96


### Statistical Analysis

Descriptive data of the performance indicators were reported as frequencies (number of observations), and percentages with an applied significance level of 5% (*p* < 0.05) was applied. Differences between categorical frequencies were determined by using chi-square. Some of the performance indicators were expressed as percentages, which according to Hughes and Bartlett (2002) provides a more accurate analysis of team performance during match-play. Six *a priori* proportions were decided upon as proxies of referee/player behavior, similar to that used by Brown et al. (2018). Referee behavior: proportion of (a) non-sanctioned illegal ruck clean outs out of all illegal ruck clean outs; (b) non-sanctioned illegal ruck clean outs out of all ruck clean outs (legal and illegal combined); (c) non-sanctioned dangerous ruck clean outs out of all illegal ruck clean outs; and (d) non-sanctioned dangerous illegal ruck clean outs out of all ruck clean outs (legal and illegal combined). Player behavior: proportion of (e) illegal ruck clean outs out of all ruck clean outs (legal and illegal combined); and (f) dangerous illegal clean outs out of all illegal ruck clean outs.

## Results

The current study revealed that 22,281 ruck clean outs occurred during the 2018 Super Rugby competition at an average of 186 cleanouts per match. The analysis revealed that 9% (*n* = 2111) of these ruck clean outs were deemed illegal according to the 2018 WR *Laws of the Game* at an average of 18 per match. The referees did not sanction 93% (*n* = 1953) of the illegal ruck clean outs at an average of 16 per match. Of the total illegal ruck cleanouts not sanctioned by the referees, 57% (*n* = 1087) were considered dangerous at an average of 10 per match.

**FIGURE 1:**
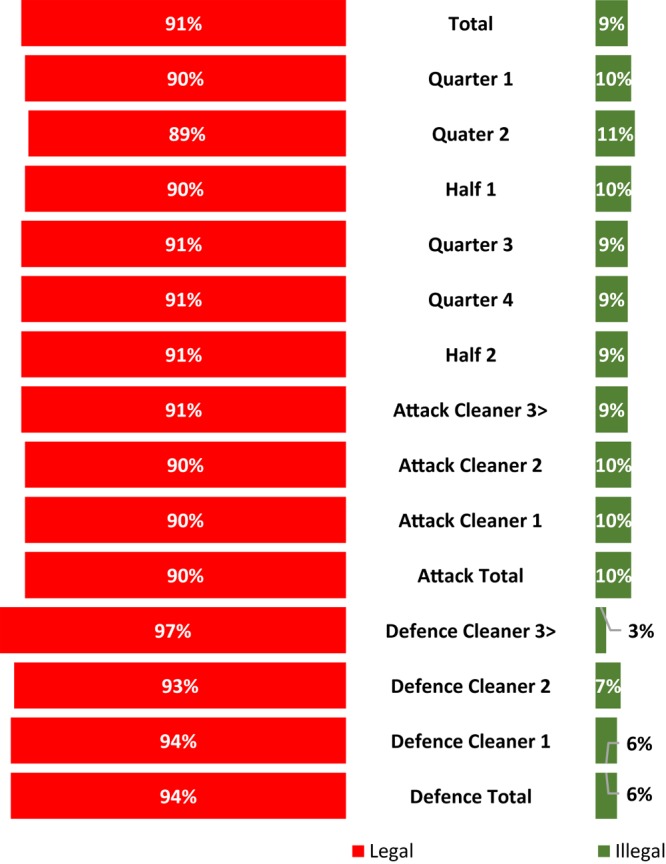
The proportion of legal (red) versus illegal (green) ruck clean outs during the 2018 Super Rugby Tournament.

The number of legal and illegal ruck clean outs coded is presented in Figure 1. The results reveal that the illegal ruck clean outs were consistently spread across the two halves and four quarters. The analysis revealed a statistically significant difference (*p* < 0.001) in the percentage of illegal clean outs between the first (11%, 1072 of 9436) and second (7%, 1038 of 10,707) halves of play. A statistically significant difference was also observed when comparing the percentage of illegal clean outs in quarter 2 (11%, 545 of 5118) and 3 (9%, 484 of 5583) (*p* < 0.001), as well as quarter 2 and 4 (9%, 555 of 6163) (*p* < 0.001). The attacking team accounted for 90% (1895 of 2111) of the total illegal ruck clean outs at an average of 16 per match. No statistically significant difference was found between the attacking and defending, or the number of cleaners, involved in the ruck cleanout. The first cleaner for both the attacking and defending team were responsible for 67% (1450 of 2111), of the illegal ruck clean outs.

**FIGURE 2:**
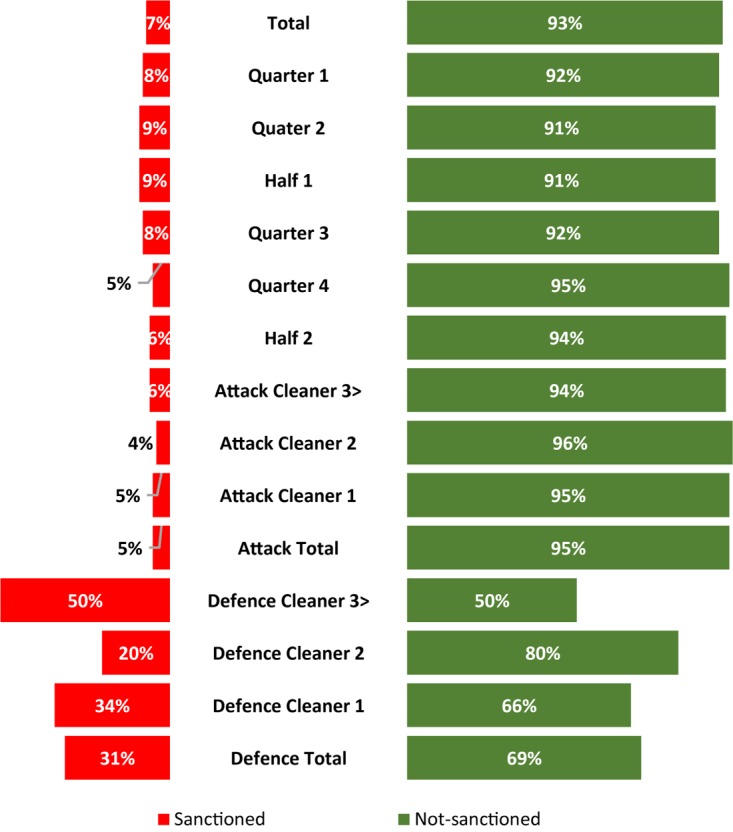
The proportion of sanctioned (red) versus non-sanctioned (green) illegal ruck cleanouts coded during the 2018 Super Rugby Tournament.

Figure 2 shows the sanctioned and not-sanctioned illegal ruck clean outs in which the referees did not sanction 93% (1953 of 2111) of total illegal ruck clean outs, presenting 9% (1953 of 22,281) of the total ruck clean outs (legal and illegal combined). The non-sanctioned illegal rucks clean outs were evenly spread across the two halves, but revealed a statistical significant (*p* < 0.05) increase from quarter 3 (*n* = 444) to 4 (*n* = 529) of the match. The referees did not sanction the attacking team for 90% (1804 out of 1953) of the illegal ruck clean outs not sanctioned. The first cleaner for both the attacking and defending team were not sanctioned for 68% (1333 of 1953) of the total illegal ruck clean outs.

Table 3 presents the non-sanctioned illegal ruck clean outs not sanctioned for both the attacking and defending team. The majority of illegal ruck clean outs not sanctioned by the referee were “*not supporting own body weight*” (32%; *n* = 624), followed by “*side entry*” (16%; *n* = 318), “*shoulder charge*” (16%; *n* = 317), and “*contact above the shoulder*” (13%; *n* = 247). The attacking team were not sanctioned when “*not supporting own body weight*” (96%; *n* = 599) and “*side entry*” (95%; *n* = 303) compared to the defending team for “*cleaning a player not involved in ruck*” (27%; *n* = 12) and “*joining the ruck from an offside position*” (12%; *n* = 22). The illegal ruck clean outs not sanctioned for the defending team were “*cleaning a player not involved in ruck*” (27%; *n* = 12), followed by “*joining the ruck from an offside position*” (12%; *n* = 9).

**Table 3:** Illegal ruck cleanouts not sanctioned for the attacking and defending team.

Type of illegal ruck cleanout	Attacking team	Defending team
	7% (*n* = 1804)	93% (*n* = 149)
Not supporting own body weight	96% (*n* = 599)	4% (*n* = 25)
Side entry	95% (*n* = 303)	5% (*n* = 15)
Shoulder charge	90% (*n* = 285)	10% (*n* = 32)
Contact above the shoulder	91% (*n* = 224)	9% (*n* = 23)
Neck roll	91% (*n* = 105)	9% (*n* = 11)
Not grasping	90% (*n* = 188)	10% (*n* = 22)
Joining the ruck from an offside position	88% (*n* = 67)	12% (*n* = 9)
Cleaning a player not involved in ruck	73% (*n* = 33)	27% (*n* = 12)


**n* = number of observations and % = percentage*.

**FIGURE 3:**
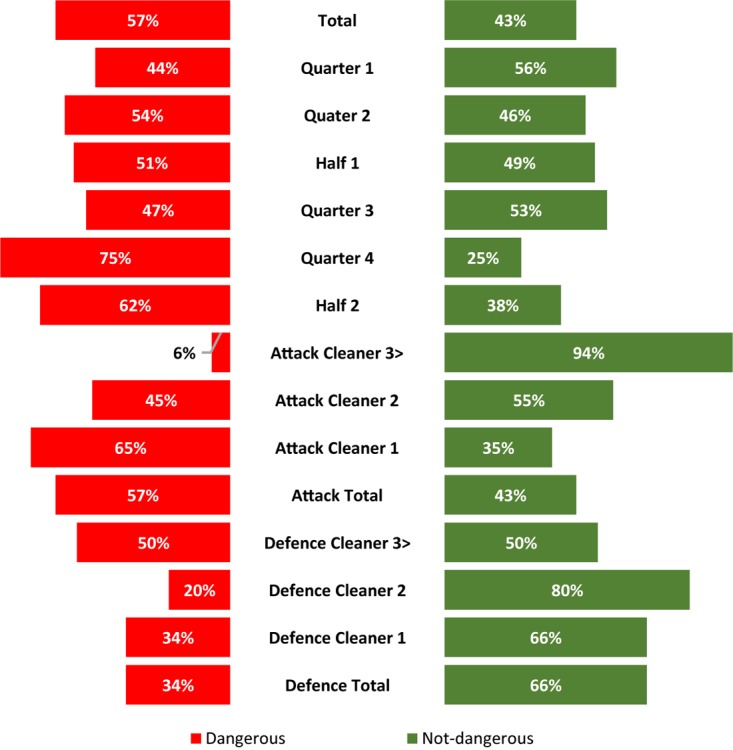
The proportion of not sanctioned dangerous (red) versus not-dangerous (green) illegal ruck cleanouts coded during the 2018 Super Rugby Tournament.

Figure 3 presents the dangerous and not-dangerous illegal ruck clean outs not sanctioned by the referee; 5% in terms of proportion (1087 of 22,281) of all the ruck clean outs and 51% (1087 of 2111), of the total illegal clean outs were deemed dangerous. The current study showed a statistical increase (*p* < 0.001) in dangerous non-sanctioned illegal ruck clean outs from the first (*n* = 481) to the second half (*n* = 606), as well as an increase from quarter 1 (*n* = 215) to 2 (*n* = 266). The same trend was observed for quarter 3 (*n* = 210) to 4 (*n* = 396). The referees did not sanction the attacking team for 95% (1037 of 1087) of the dangerous illegal ruck clean outs. The first cleaner for the attacking team was not sanctioned 77% (801 of 1037) of the dangerous illegal ruck clean outs.

Table 4 presents the dangerous and not dangerous illegal ruck cleanouts not sanctioned by the referees. The majority of dangerous illegal clean outs not sanctioned were “*shoulder charge*” (88%, *n* = 280), followed by “*neck roll*” (86%, *n* = 100) and “*contact above the shoulder*” (81%, *n* = 201).

**Table 4:** Dangerous and not dangerous illegal ruck cleanouts not sanctioned.

Type of illegal ruck cleanout	Dangerous	Not dangerous
	57% (*n* = 1087)	43% (*n* = 866)
Shoulder charge	88% (*n* = 280)	12% (*n* = 37)
Neck roll	86% (*n* = 100)	14% (*n* = 16)
Contact above the shoulder	81% (*n* = 201)	19% (*n* = 46)
Not supporting own body weight^∗^	64% (*n* = 280)	36% (*n* = 344)
Side entry^∗^	60% (*n* = 190)	40% (*n* = 128)
Cleaning a player not involved in ruck^∗^	22% (*n* = 10)	78% (*n* = 35)
Not grasping^∗^	10% (*n* = 20)	90% (*n* = 190)
Joining the ruck from an offside position^∗^	8% (*n* = 6)	92% (*n* = 70)


*^∗^the first infringement by the cleaner was not deemed dangerous but the action by the cleaner that followed the initial cleanout was deemed dangerous; *n* – number of observations and % – percentage*.

## Discussion

The major findings of this study were that: (a) on average 57% (*n* = 1 087) of all the dangerous illegal ruck clean outs were not sanctioned by the on-field referees according to the 2018 WR *Laws of the Game*; (b) the on-field referees did not sanction the attacking team for 95% (1037 out of 1087) of all the dangerous illegal ruck clean outs; and (c) the majority of the dangerous illegal clean outs not sanctioned by the referees were “*shoulder charge*,” “*neck roll*,” and “*contact above the shoulder*.” To the researchers’ knowledge, this is the first study that investigated non-sanctioning of dangerous illegal clean outs at the ruck by on-field referees during match play. Other studies investigated the non-sanctioning of illegal tackles in professional (Fuller et al., 2010) and youth (Brown et al., 2018) rugby. The findings of the present study are a concern for rugby referee stakeholders from an error rate perspective as 1953 non-sanctioned illegal ruck clean outs were identified. A greater concern for rugby safety and rugby referee stakeholders should be that 1087 of these non-sanctioned ruck clean outs were deemed dangerous, which could pose an injury risk to the players involved in the ruck area.

The rate of illegal and dangerous illegal ruck clean outs were considered high: 9 and 5%, respectively, of the ruck clean outs, this finding does not bode well for player behavior at elite level. Furthermore, when the errors made by the on-field referees were contextualized in comparison to the total number of ruck clean outs, it will be difficult for referees to detect and sanction all illegal ruck clean outs due to the number of players involved. However, from an injury prevention perspective, referees can minimize the risk by focusing on “*shoulder charge*,” “*neck roll*,” and “*contact above the shoulder*,” given the high proportion of these infringements that are deemed dangerous. While the rate of non-sanctioned illegal ruck and non-sanctioned dangerous illegal ruck clean outs to all the ruck clean outs could be seen as a proxy for referee behavior, the rate of illegal and dangerous illegal ruck clean outs to all the ruck clean outs in turn can be seen as a metrics of player behavior during match-play.

The high error rate by the referees for illegal and dangerous illegal ruck clean outs (especially the first cleaner for both the attacking and defending team) in the current study could be due to the positioning of the referee at the ruck, which influences whether there is a clear view of the clean out for both the attacking and defending players. The study by Kraak et al. (2011) revealed that the on-field referees movement patterns varied per game due to (a) referee experience – during the study the experienced referees moved less because they anticipate subsequent play better than the novice referees; (b) the level or/and quality of the competition that are being observed; (c) the intensity and different match-play activities completed by the attacking and defending players; (d) the referees time of arrival at the ruck; and (e) application of the laws – Spitz et al. (2016) stated that perceptual and cognitive skills are required by on-field referees to make sure that the decision-making process results in accurate, consistent, and uniform decisions, (f) decision-making (application of the law) ability by the on-field referee. Wheeler et al. (2013) indicated that it is difficult for on-field referees to officiate and sanction the ruck area accurately due to the number of attacking and defending players involved in this phase of play. According to Souchon et al. (2010) on-field referees are faced with making complex decisions in limited time, research has shown that on-field referees rely on judgmental heuristics (i.e., quick and easy decision laws), to help them make their decisions. Research by Mascarenhas et al. (2005) in rugby and Hancock and Ste-Marie (2013) in ice hockey attempted to quantify referee decisions during match play. The rugby on-field referees were accurate in their sanctioning 50% of the time compared to the 75% accuracy of the ice hockey officials (Mascarenhas et al., 2005; Hancock and Ste-Marie, 2013). Although video-based assessments could provide valid examination of decision-making performance by on-field referees, they do not replicate the physical, physiological, and psychological aspects of the match (Emmonds et al., 2015). The lowest accuracy in sanctioning was observed during the last quarter of the match, advising that physical and psychological fatigue could occur during the final stage of the match (Emmonds et al., 2015). This is supported by the fact that the referees had higher error rates when players were going off their feet, joining from the side and charging into the ruck with the shoulder. The referee can easily miss these infringements because of poor positioning. The current study revealed that referees were not consistent with the application of the laws for the attacking and defending team because the referees favored the attacking team more. According to Kraak et al. (2016, 2017), a possible reason why referees tend to favor the attacking team, by penalizing the defending team more frequently, was to improve the continuity of the game. If more of these dangerous illegal ruck clean outs were picked up by referees and sanctioned accordingly, it might improve player behavior and subsequently reduce the number of dangerous illegal ruck clean outs. Therefore, the potential knock-on effect of stricter application of the laws and minimizing the sanctioning error rates of referees should not be ignored (Brown et al., 2018).

The findings of the current study showed that the attacking teams arriving players will engage in an illegal clean out in order to retain possession of the ball more frequently. A typical game situation on attack can be as follows: after the initial collision and placement by the ball carrier, the first attacking arriving player has to clear the first defender away from the ball carrier and then the second attacking arriving player secures the possession along with engaging the additional defenders as they arrive to support the first defender (Kraak and Welman, 2014). A possible reason for the high error rate by the attacking team could be because of: (a) the ball carrier is not dominating the collision and is not presenting the ball in an effective position (Hendricks et al., 2018), therefore, the arriving player must use an illegal technique to try and retain possession, (b) the attacking teams arriving players reaction time is poor from the prior activity, and thus, arrive late at the collision, (c) poor decision-making and assessment of the situation, (d) poor ruck cleaning techniques used in the latter period of the match due to fatigue (Burger et al., 2018), and (e) the defending team might be infringing already because the attacking team has no other option but to use illegal techniques in order to retain possession. Based on studies by Wheeler et al. (2010) and Kraak and Welman (2014), it is obvious that players have to execute specific actions and techniques to retain (attacking team) or regain (defending team) possession of the ball at the ruck.

The current study further identified a need for coaches and trainers to equip themselves with information pertaining to safe and effective techniques. The coaches and trainers should emphasize the importance of safe and effective techniques during training and matches and one of the few possible modes to reduce injuries, especially non-fatal catastrophic injuries toward the head, neck, brain, and spine (Posthumus and Viljoen, 2008; Brukner and Khan, 2012). The ruck is a dynamic situation, and therefore, coaches should not coach the ruck clean out in isolation because this limits the decision-making ability of the players. Ruck drills should include the initial tackle, fight for dominance by the ball carrier, placement of the ball, and clearing techniques in the same drill because this will assist players with adjustment and decision-making based on the situation. For example, after demonstrating the techniques to carry the ball effective into contact, the ball carrier should also be expected to fall sideward (away from the defending team cleaners), and present the ball (Hendricks et al., 2018), which is followed by the arriving player(s) who clean out the ruck based on the situation. These safe and effective techniques should be incorporated and emphasized during training in order to prepare players for matches. Coaches should also invite referees to training to officiate contact training sessions/drills according to the laws of the game, which will provide clarity to both players and coaches with input from the referees.

It, therefore, seems clear that referees, coaches and players play an integral part in cleaning up the ruck area and so additional strategies aimed at these role players might be beneficial to further reduce the risk of injury at this phase of play. This perspective is also consistent with the BokSmart’s adopted goal of “Vision Zero” which try to eliminate all serious injuries from the game (Brown et al., 2017).

### Practical Application

The findings of this study provide referees with an understanding of the importance of the decision-making process during match-play, especially at the rucks area. On-field referees should be firmer when it comes to discipline at the rucks because illegal dangerous ruck clean outs could result in serious injuries for players. Rugby stakeholders should also consider using two referees during a game as is currently being used by Rugby League and residence rugby union at Stellenbosch University, South Africa. The study further provides players, coaches, and rugby stakeholders with data that suggests that the ruck area needs to be more seriously recognized when it comes to injury prevention. Player behavior plays a large role regarding discipline and executing of safe and effective cleanout technique at the ruck. They must recognize their mistakes and illegalities and take responsibility for their actions. The ability of a player to engage and tolerate frequent contact events such as the ruck, whether as a ball-carrier, tackler, or attacking or defending cleaner influences the performance of the team and exposes the players to a high risk of injury. Furthermore, these safe techniques need to be added to coaching manuals for the ruck cleanout. Additional interventions need to be targeted at referees to improve identification according to the WR *Laws of the Game* and appropriate sanctioning of illegal ruck clean outs whether dangerous or not.

## Conclusion

This study of referee sanctioning in the 2018 Super Rugby competition found that 91% of the ruck clean outs were legal and 9% illegal according to the 2018 WR *Laws of the Game*. Of the illegal ruck clean outs 93% (*n* = 1953) were not sanctioned by the referees of which 57% (*n* = 1087) were deemed dangerous. The attacking team were not sanctioned 92% (*n* = 1804) for illegal ruck clean outs. The first cleaner for both the attacking 68% (*n* = 1224) and defending 73% (*n* = 109) teams contributed to the majority of the illegal ruck clean outs that were not sanctioned by the referees. The majority of dangerous illegal ruck clean outs not sanctioned by the referees were “*shoulder charge*” (88%, *n* = 280), “*neck roll*” (86%, *n* = 100), and “*contact above the shoulder*” (81%, *n* = 201). Similar to the study by Brown et al. (2018), the current study was not designed to identify factors associated with non-sanctioning behaviors of on-field referees during match-play. The fact that on-field referees error rates did not change as the match progressed, suggests that the primary cause could not be associated with fatigue. What should be highlighted is that there was an increase in dangerous illegal ruck clean outs not sanctioned in the second half, quarter 2 and 4 of the match. However, to aid injury prevention efforts, future studies should explore why referees are not sanctioning all illegal ruck clean outs as per the WR *Laws of the Game*. Additional interventions like referee decision-making and specific fitness interventions need to be targeted at referees to improve this shortcoming. The results from this study highlight many areas for potential research. Future studies should investigate the non-sanctioning of illegal ruck clean outs in other elite competitions, as well as at the amateur level and should also include factors like zonal locations, score line, log position, and nationality of teams. Furthermore, studies should also focus on the behavioral aspects regarding the player’s discipline toward technique used for cleaning out at the ruck and education of coaches.

## Author Contributions

WK was the leader author, responsible for data collection, and conceptualized the research idea with JB, SK, SH, and UJ. JB, SK, SH, and UJ were responsible for exporting the raw data from the Nacsport Basic+ software into Excel and conducted the literature search for the study. KS provided expert advice on the injury prevention and safety section of the article. All the authors were responsible for drafting and approval of the final manuscript.

## Conflict of Interest Statement

The authors declare that the research was conducted in the absence of any commercial or financial relationships that could be construed as a potential conflict of interest.
